# Comparing the Mechanical Properties of Rice Cells and Protoplasts under PEG6000 Drought Stress Using Double Resonator Piezoelectric Cytometry

**DOI:** 10.3390/bios14060303

**Published:** 2024-06-09

**Authors:** Yu Yan, Tiean Zhou, Yu Zhang, Zhicheng Kong, Weisong Pan, Chengfang Tan

**Affiliations:** 1 College of Bioscience and Biotechnology, Hunan Agricultural University, Changsha 410128, China; yanyu9778@163.com (Y.Y.); joux19@163.com (W.P.);; 2 Hunan Provincial Engineering Technology Research Center for Cell Mechanics and Function Analysis, Changsha 410128, China

**Keywords:** double resonator piezoelectric cytometry, drought stress, rice cell, protoplasts, cells generated stress, cell viscoelasticity

## Abstract

Plant cells’ ability to withstand abiotic stress is strongly linked to modifications in their mechanical characteristics. Nevertheless, the lack of a workable method for consistently tracking plant cells’ mechanical properties severely restricts our comprehension of the mechanical alterations in plant cells under stress. In this study, we used the Double Resonator Piezoelectric Cytometry (DRPC) method to dynamically and non-invasively track changes in the surface stress (ΔS) generated and viscoelasticity (storage modulus G′ and loss modulus G″) of protoplasts and suspension cells of rice under a drought stress of 5–25% PEG6000. The findings demonstrate that rice suspension cells and protoplasts react mechanically differently to 5–15% PEG6000 stress, implying distinct resistance mechanisms. However, neither of them can withstand 25% PEG6000 stress; they respond mechanically similarly to 25% PEG6000 stress. The results of DRPC are further corroborated by the morphological alterations of rice cells and protoplasts observed under an optical microscope. To sum up, the DRPC technique functions as a precise cellular mechanical sensor and offers novel research tools for the evaluation of plant cell adversity and differentiating between the mechanical reactions of cells and protoplasts under abiotic stress.

## 1. Introduction

Water deficit has a major effect on rice, an important cereal crop. In response to drought stress, proline and glycine levels in plant cells rise and influence cellular metabolism [1]. The dynamics of the cell wall are crucial when drought stress is applied to rice cells [2,3], particularly in the processes of cell wall remodeling and synthesis. Research shows that plants can thicken their cell walls during drought stress by depositing lignin and hemicellulose, which improves secondary walls [4,5,6]. Furthermore, cell wall flexibility may rise or fall as a result of dryness [7,8]. Cell wall elasticity and drought tolerance were found to be correlated in studies comparing elastic moduli under drought stress in six types of radiata pine and common bean [9,10]. Increased peroxidase activity and cell wall hardening in rice seedling roots may be related, according to research by Chuan Chi Lin and colleagues [11]. The plasma membrane is an excellent medium for detecting and transferring mechanical pressures because it is located between the cytoskeleton and the cell wall [12,13]. When plants experience drought stress, their plasma membrane’s composition varies and its integrity is compromised [14,15]. Plant growth and environmental adaptability depend on the dynamic plant cytoskeleton’s ability to quickly modify tissue stability and dynamics in response to both internal and external stimuli [16]. Actin filaments and microtubule proteins display depolymerization and polymerization processes when subjected to drought stress. Actin filaments (AFs) and microtubules (MTs) control cell rigidity and energy dissipation [17]. Moreover, once they become active, early indicators of drought stress such as Ca^2+^, reactive oxygen species (ROS), ion channels, kinases, and phosphatases [18] may cause structural and functional modifications to the cytoskeleton, plasma membrane, and cell wall, changing the mechanical characteristics of plant cells. Unfortunately, there are not many effective detection methods available for following the dynamic changes in the mechanical properties of plant cells right now. Plant cells would maintain mechanical homeostasis, or “mechanostasis”, wherein the strength of the cell wall and the magnitude of turgor are in balance [19]. During cell growth or under abiotic stress, both a cell wall’s mechanical properties and turgor would either maintain a new mechanostasis or undergo adaptive changes.

However, it is experimentally challenging to track and separate the effects of turgor and a cell wall’s mechanical properties. In particular, under abiotic stress, a cell would lose its turgor and generate a negative turgor pressure [20]. There is no appropriate method to measure negative turgor pressure at the cellular level. The hydrostatic pressure (or turgor pressure *P*) is related to the tensile stress (σ) generated within the wall for an idealized spherical plant cell according to Equation (1) [21].

(1) $$\sigma =\frac{PR}{2h}\;\Leftrightarrow \;P=\frac{2h\sigma }{R}$$

where *R* and *h* are the radius of the cell and thickness of the cell wall, respectively. There is currently no method available to measure either turgor pressure *P* or tensile stress (σ) in a non-invasive and continuous manner (Table 1 [22,23,24,25,26,27,28]). Thus, there is a great need to develop a new technique to monitor the changes in cells’ generated forces and viscoelasticity of plant cells under development and various stresses.

A technique based on quartz crystal for measuring minute masses is called QCM (Quartz Crystal Microbalance). QCM sensors are characterized by their high sensitivity, non-destructiveness, and real-time measurements [26]. QCM was used by Mei Zeng et al. [29] to track the dynamic processes of rice cell adhesion to PLL-poly (3-PBA)/Au and salt stress treatment with NaCl in real time. QCM-D technology was employed by Chen et al. [30] to track the dynamic viscoelastic alterations of tobacco BY-2 cells at varying mannitol concentrations. However, these studies only provided semi-quantitative information about cells’ viscoelasticity by using cell viscoelastic index (CIV), and no information about cellular force was given. Recently, we developed the Double Resonator Piezoelectric Cytometry (DRPC) technique [31], which monitors and measures the forces generated by cells and cells’ viscoelastic moduli using two different crystals of AT and BT cuts with the same frequency and surface morphology.

Our team has applied the DRPC technique for tracking the dynamic adhesion of H9C2 cells and evaluating the two cardiovascular medications on H9C2 cardiomyocytes’ contractile properties [32] for distinguishing between the necrosis and apoptosis of HeLa cells based on their respective cyto-mechanical characteristics [33]. Polyethylene Glycol (PEG) is a non-ionic, water-soluble polymer that is considered safe in terms of toxicity. PEG can be used to alter the osmotic potential of the culture medium, inducing plant dehydration in a relatively controlled manner in experimental settings [34]. Additionally, PEG with a high molecular weight (≥60,000) does not penetrate cell walls, making it an ideal material for simulating drought stress in plant cells and recreating soil-like conditions in vitro. Currently, PEG6000 has been widely used to simulate the effects of drought stress on plants in various aspects such as biochemical, physiological, metabolic, and photosynthetic studies [35,36]. In this work, DRPC chips were used to monitor the dynamic changes in cellular forces and cells’ viscoelastic moduli of both rice cells and rice protoplasts in real time under the treatments of varying concentrations of PEG6000 to simulate drought stress. This research explored the possibilities of using the DRPC technique in studying the dynamic changes in cell wall–plasma membrane–cytoskeleton continuum and is expected to offer a novel research tool for investigating the stress resistance of plant cells.

## 2. Materials and Methods

### 2.1. Materials and Major Reagents/Instruments

**Figure 1:**
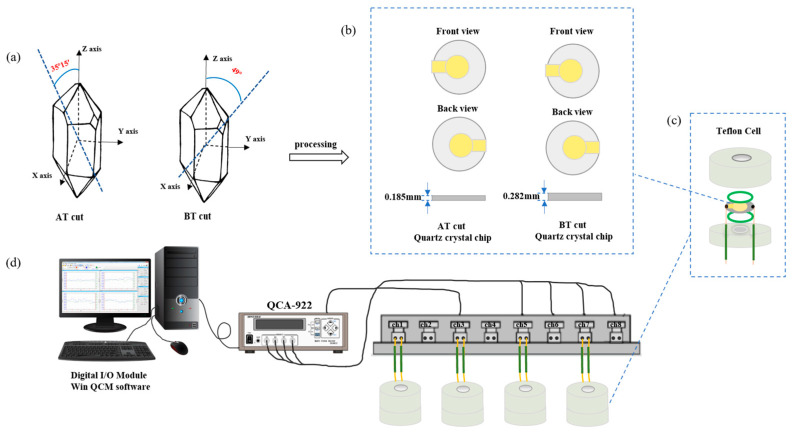
Schematic illustrations of AT- and BT-cut crystals and the DRPC-based experimental setup. (**a**): quartz crystal orientations, (**b**): AT-cut and BT-cut chips, (**c**): Teflon well assembly, (**d**): DRPC setup.

Rice Material: Seeds of *Oryza sativa* L. *japonica* cv. Experimental apparatus: QCA922 eight-channel quartz crystal microbalance (Seiko-EG&G, Tokyo, Japan); AT and BT-cut crystals of 5 MHz and 9 MHz with gold electrodes were used in the experiments; the quartz crystal blanks were made by Hangzhou Zhongjing Electronic Technology Co., Ltd. (Hangzhou, China), and the gold coatings were deposited by Beijing Chenjing Electronic Co., Ltd. (Beijing, China). Figure 1 shows the schematic illustrations of AT- and BT-cut crystals and the DRPC-based experimental setup. AT and BT-cut quartz crystals were cut at angles of 35°15′ and −49° relative to the crystallographic Z axis, respectively (Figure 1a). The crystals were then machined to the required thickness and sizes. The thicknesses of the 5 MHz and 9 MHz AT- and BT-cut crystals were 0.333 mm (5 MHz, AT), 0.510 mm (5 MHz, BT), 0.185 mm (9 MHz, AT), and 0.282 mm (9 MHz, BT). The crystals were wafers of a 13.67 mm diameter, which were deposited with a 10 nm chromium adhesion layer followed by the deposition of 100 nm gold of a 5.5 mm diameter (Figure 1b). Each of the AT- and BT-cut crystals was sandwich-assembled in a Teflon well by two silicon O-rings with screws (Figure 1c). Two wires bonded to the flag parts of the gold electrodes of the crystal using conducting paste were connected to an electrical socket of the QCA 922-90 multiplexer, which was connected to a QCA 922 Quartz Crystal Analyzer (Seiko-EG&G, Tokyo, Japan) via an RS-232 interface (Figure 1d); this unit allows us to continuously measure a maximum of eight channels of a crystal’s frequency and resistance signals by Win QCM software (version 2.2.0.0)

### 2.2. Sample Preparation

Preparation of Rice Suspension Cells: A plant incubator was set to a dark temperature of 25 °C and 60% humidity. The seeds were sterilized and injected onto a solid culture medium. Following the development of callus tissue and the enlargement of the lower hypocotyl, the callus tissue was moved to a liquid culture medium and positioned in a shaking bed with a constant temperature of 25 °C and a rotation speed of 130 rpm/min for dark cultivation. Before being used, the cells were passed through a 300–400 mesh screen and then counted.

Preparation of Rice Protoplasts: After the rice suspension cells were prepared as previously described, they were put in a confocal glass dish and agitated at a rate of 60 rpm/min while being treated to a dark enzymatic digestion at 25 °C. A large number of fully rounded rice protoplasts were revealed by a microscope after around five hours, after being passed through a 400–500 mesh sieve.

### 2.3. Quantitative Measurements of Surface Stress Generated by Plant Cells and their Viscoelastic Moduli using DRPC Technology

A detailed theoretical background and methods of DRPC are introduced in our previous publication [31]. It is based on the acoustic wave principle that the surface stress exerted on the AT and BT chips would affect the elastic moduli, which are related to their second-order and third-order elastic constants as well as the cutting angle φ (angle of rotation about the crystallographic x-axis) and, therefore, their frequencies. The cells’ viscoelastic parameters are then calculated by treating the viscoelastic load as an additional acoustic impedance by measuring changes in the crystals’ frequency and motional resistance or bandwidth. Here, we briefly introduce the DRPC technique and its extension for the study of plant cells. The frequency change Δf caused by cell adhesion and various stimuli is typically in the range of a few hundred Hz, which is much smaller than the frequency f_0_ of the quartz crystal (typically 5 MHz or 9 MHz), i.e., Δf/f_0_ << 1. Consequently, the observed total relative frequency shift can be expressed by the following linear perturbations of the quartz crystal: cell’s generated surface stress, cell mass, and viscoelasticity.

(2) $$\Delta f/f_{0}=\Delta f_{s}/f_{0}+\Delta f_{m}/f_{0}+\Delta f_{visco}/f_{0}$$

Because the shear waves’ penetration depth to the cells is much smaller than that of the cells itself, cells can be thought of as a semi-infinite viscoelastic load. The following equations are relative frequency shifts induced by surface stress cells exerted on the quartz crystal chips (ΔS), mass of the cells (Δm), and cells’ viscoelastic parameters; while K and µ*_q_* are stress coefficient and elastic modulus, both of which are cut-dependent. ρ*_q_* is quartz density, which is cut-independent, and Z*_q_*=$\sqrt{\rho_{q}{µ}_{q}}$ is the acoustic impedance of the quartz.

$$\frac{\Delta f_{s}}{f_{0}}=\frac{K\Delta S}{t_{q}},\frac{\;\Delta f_{m}}{f_{0}}=-\frac{2f_{0}\Delta m}{\sqrt{\rho_{q}\mu_{q}}},\frac{\Delta f_{visco}}{f_{0}}=-(\pi Z_{q}{)}^{-1}\sqrt{\rho_{c}\left(\left|G^{*}\right|-G^{\prime }\right)/2}$$

Therefore, Equation (2) can be expressed as

(3) $$\Delta f/f_{0}=K\Delta S/t_{q}-(\sqrt{\rho_{q}\mu_{q}}\;{)}^{-1}\left(2f_{0}\Delta m+\pi^{-1}\sqrt{\rho_{c}\left(\left|G^{*}\right|-G^{\prime }\right)/2}\right)$$

Applying Equation (3) to AT and BT cuts, we have

(4) $$\Delta f^{AT}/f_{0}^{AT}=K^{AT}\Delta S/t_{q}^{AT}-(\sqrt{\rho_{q}{\mu_{q}}^{AT}}\;{)}^{-1}\left(2f_{0}^{AT}\Delta m+\pi^{-1}Im(\sqrt{\rho_{c}\left(G^{\prime }+iG”\right)}\right)$$

(5) $$\Delta f^{BT}/f_{0}^{BT}=K^{BT}\Delta S/t_{q}^{BT}-(\sqrt{\rho_{q}{\mu_{q}}^{BT}}\;{)}^{-1}\left(2f_{0}^{BT}\Delta m+\pi^{-1}Im(\sqrt{\rho_{c}\left(G^{\prime }+iG”\right)}\right)$$

Given that the assumed frequencies of AT-cut and BT-cut crystals are equal, f_0_^AT^ = f_0_^BT^ = f_0_, and taking into account the correlation between quartz crystal thickness t*_q_*, crystal frequency, and elastic modulus, we have:

(6) $$\;t_{q}=0.5f_{0}^{-1}{(\rho }_{q}/\mu_{q}{)}^{-0.5}$$

Combining Equations (4)–(6) and under the condition of f_0_^AT^ = f_0_^BT^ = f_0_, the contributions of cells’ mass and viscoelastic terms of Equations (4) and (5) can be canceled, resulting in Equation (7), illustrating the dynamic change in surface stress exerted by the cells.

ΔS_t_= f_0_^−1^(K^AT^ − K^BT^)^−1^(Δf_t_^AT^t_q_^AT^ − Δf_t_^BT^t_q_^BT^) (7)

Here, the stress coefficients of AT-cut and BT-cut quartz crystals are K^AT^ = 2.75 × 10^−l2^ cm^2^ dyn^−1^ and K^BT^ = −2.65 × 10^−l2^ cm^2^ dyn^−l^, respectively; the thicknesses of AT-cut and BT-cut quartz crystals, t*_q_*^AT^ and t*_q_*^BT^, are both related to the frequencies of AT- and BT-cut crystals; f_0_ is the resonant frequency of AT-cut and BT-cut quartz crystals; Δf_t_^AT^ and Δf_t_^BT^ are the frequency shifts of AT-cut and BT-cut quartz crystals with respect to their reference points (stable values in the culture medium) at any given time (t).

When f_0_^AT^ = f_0_^BT^ = 9 MHz, t*_q_*_9M_^AT^ = 0.185 mm, t*_q_*_9M_^BT^ = 0.0282 mm, Equation (7) can be simplified to

ΔS = 380.8Δf^AT^ − 582.2Δf^BT^ (8)

When f_0_^AT^ = f_0_^BT^ = 5 MHz, t*_q_*_5M_^AT^ = 0. 333 mm, t*_q_*_5M_^BT^ = 0.510 mm, Equation (7) can be simplified to

ΔS = 1230.4Δf^AT^ − 1878.5Δf^BT^ (9)

Hz is the unit of Δf^AT^ and Δf^BT^, while dyne/cm is the unit of ΔS. When ΔS = 0, according to Equation (1) of the manuscript, the turgor pressure P = 0 where incipient plasmolysis occurs, and the cells are in their equilibrium state. When ΔS is positive, it means that the cells are enlarged and they are in a tensile state (e.g., under hydrostatic pressure or turgor pressure). When ΔS is negative, the plant cells are in a contractile state and they are producing compressive stress. As far as the ranges of positive and negative ΔS, it is known [20] that at least three factors of cell size, cell age, and ‘sclerophylly’ (stiffness) would affect the largest negative range the cells can endure. For example, a small cell size permits plants to endure negative values of water potential with relatively little water loss. Structurally, the largest negative ΔS value the cells can endure depends on if the integrity of the cell wall–plasma membrane–cytoskeleton continuum could be maintained. In particular, the breakage of the Hechtian strands would lead to irreversible damage of the cells. For wall-free protoplasts, the largest negative ΔS would be the state when the cytoskeleton structures are irreversibly damaged and cannot be recovered. Theoretically, the largest positive ΔS value is when the cells are in pure water with the largest turgor pressure; however, the cells may not be able to maintain turgor pressure when the osmotic pressure decreases to some degree as we observed in some cells under hypotonic conditions. For protoplasts, the cells may be broken even in pure water. The above Equations (7)–(9) are derived under the conditions that the cells’ mass, viscoelasticity, and generated surface stress are constantly changing, whereas the cells’ medium is maintained unchanged. However, during the abiotic stresses, the density, viscosity, and viscoelasticity of the media would change depending on the type of stress. For drought stress as simulated by PEG6000 solutions, the viscoelasticity of the media would change. In this case, an additional term characterizing the viscoelasticity of PEG6000 can be added to the inside of the second brackets of Equations (4) and (5); eventually, it would still produce the same equations as Equations (7)–(9).

Furthermore, real-time cell viscoelasticity monitoring is possible using the DRPC approach. The frequency shift Δ*f* subtracted from the total frequency shift from the contribution induced by surface stress, change in either half-bandwidth (Γ), or motional resistance (Δ*R*) relative to their values in air can be used to determine the storage modulus (G′) and loss modulus (G″) of the cells. Specific relationships are as follows:

(10) $$G\prime =\frac{\pi^{2}{Z_{q}}^{2}}{\rho_{c}{f_{0}}^{2}}(\frac{\Delta R^{2}}{16\pi^{2}L_{q}^{2}}-∆f^{2})$$

(11) $$G\prime \prime =-\frac{{\pi Z_{q}}^{2}\Delta f\Delta R}{2\rho_{c}L_{q}{f_{0}}^{2}}$$

The quartz crystal’s impedance, *Z_q_*, is 8.84 × 10^5^ g/cm⋅s for AT-cut crystal, *ρ_c_* is the cells’ density, which is taken to be the same as that of water. *L_q_*, which is regarded as a constant, is the quartz crystal’s measured inductance while submerged in the medium.

### 2.4. Dynamic Monitoring of the Mechanical Properties of Rice Cells under Different Concentrations of PEG6000 Stress Using DRPC

Here, 9 MHz AT-cut and BT-cut chips were cleaned by following the procedure described by Mei Zeng et al. [29], and then the chips were surface-modified with 1% PDADMAC at 25 °C for 30 min under dark conditions, followed by rinsing with deionized water and drying with nitrogen gas. The modified chips were assembled with cleaned Teflon pools to form the detection chambers, and 500 μL of the medium was added to each of the chambers. The detection chambers were connected to a QCA-922 instrument to monitor the changes in frequencies and motional resistances of the 9 MHz AT-cut and BT-cut chips. Once the frequency and resistance values became stable, 200 μL of a mixture of culture medium containing 50,000 cells was added to each of the chambers, and the data were recorded at this stage for 3 h by the instrument. Then, the PEG6000 stock solution was added to the chambers to achieve a volume percentage of 5%, 10%, 15%, and 25% of PEG6000 in the system, respectively, and the changes in resistance and frequency were continuously monitored. The control group was the same but without adding the rice cells, and it followed the same steps as the experimental group.

### 2.5. Dynamic Monitoring of the Mechanical Properties of Rice Protoplasts under Different Concentrations of PEG6000 Stress Using DRPC

In the experiment involving rice protoplasts, 5 MHz chips were used as the substrates for the attachments of the protoplasts, and all other experimental steps were the same as in Section 2.4.

### 2.6. Microscopic Observations of the Morphologies of Rice Cells and Protoplasts under Different Concentrations of PEG6000 Stress

To observe the cells’ morphologies at different concentrations, cells or protoplasts were attached to a 12-well plate following the same method as described in Section 2.4. Solutions of PEG6000 ranging from 0% to 25% (2 mL) were added to each well. The plates were kept at 25 °C in the dark for 1 h, and the cells’ morphologies at each concentration were observed using a Lumascope 720 microscope.

## 3. Results

### 3.1. The Mechanical Changes of Rice Cells under Different Concentrations of PEG6000 Stress Concentrations

**Figure 2:**
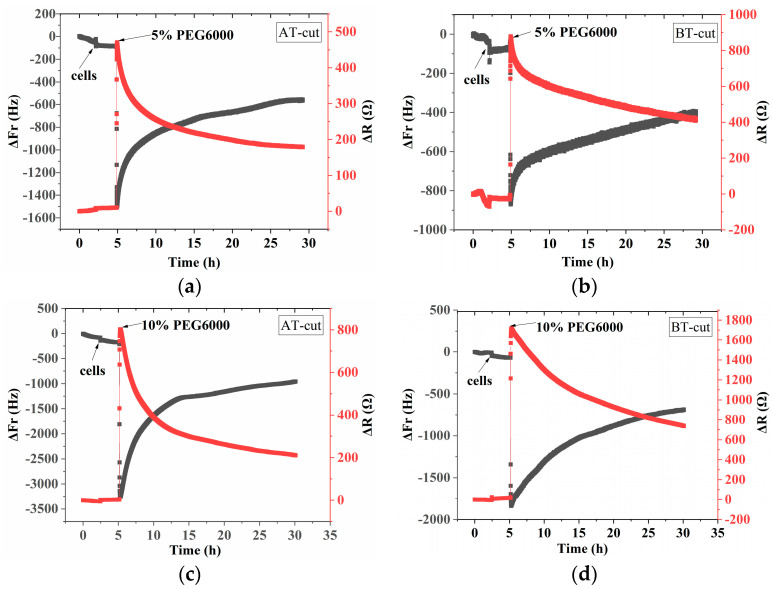

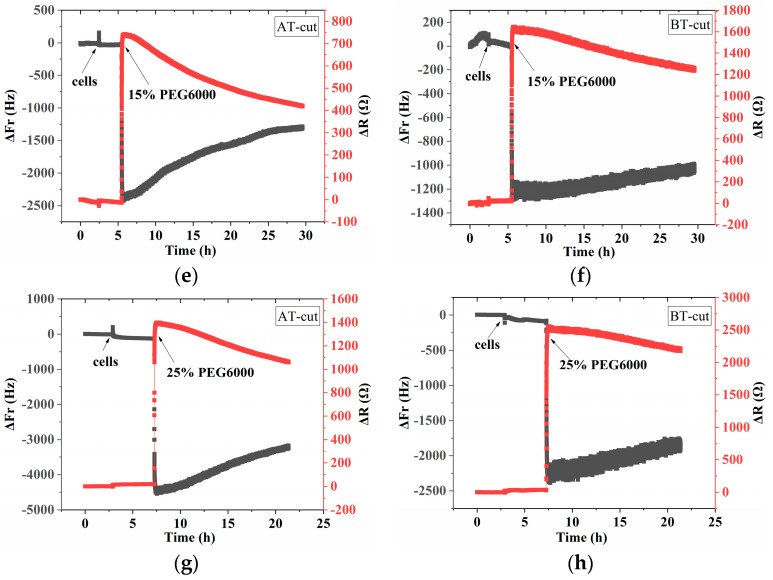
9 MHz DRPC tracking of frequency−resistance alterations brought about by rice cells under varying PEG6000 stress concentrations: 5% PEG6000 in (**a**,**b**), 10% PEG6000 in (**c**,**d**), 15% PEG6000 in (**e**,**f**), and 25% PEG6000 in (**g**,**h**).

**Figure 3:**
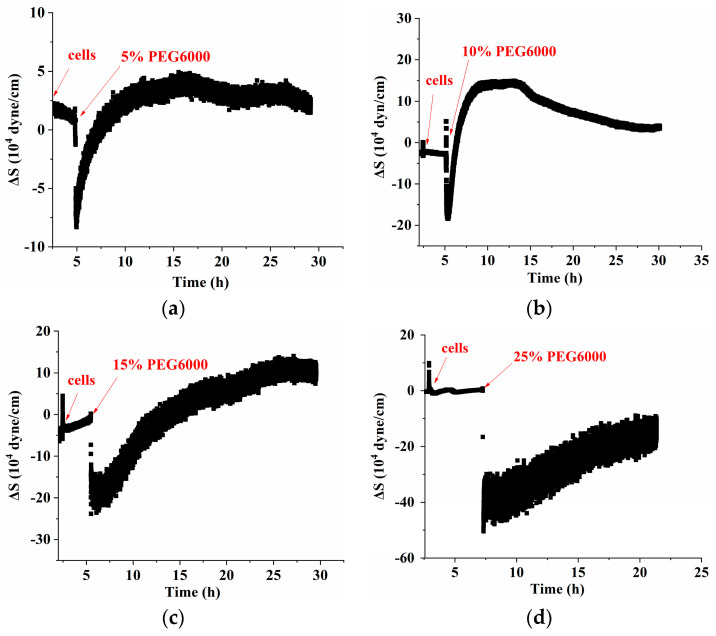
The 9 MHz DRPC tracking of stress (ΔS) variations produced by rice cells under various PEG6000 stress concentrations. PEG 6000 percentages are as follows: (**a**) 5%, (**b**) 10%, (**c**) 15%, and (**d**) 25%.

Figure 2 shows the frequency and resistance changes of AT- and BT-cut quartz crystal chips during the adhesions of rice cells followed by the treatments of different PEG6000 stress concentrations. Upon the introduction of roughly 50,000 cells, ΔF decreased and ΔR increased as the cells adhered to the quartz crystal chip. The cells induced DRPC chips’ responses, showing a sudden decrease in ΔF followed by an increase, and a sharp increase in ΔR followed by a decrease, upon the addition of 5–25% PEG6000 stresses. Moreover, the variations in ΔF and ΔR progressively increased as the concentration rose. The initial sudden drop and increase in ΔF and ΔR, respectively, is a strong indication of the increased adhesion of the cells to the chips due to contraction. Following this initial period, dramatic rebounds of both ΔF and ΔR appeared; this can be ascribed to the contraction of protoplast from the cell wall. The stress (ΔS) produced by the cells under a 5–25% PEG6000 stress can be calculated using Formula (8) in accordance with the DRPC [31] principles, as seen in Figure 3. The cells exerted compressive stress first, followed by tensile stress, as seen by the rapid rebound of ΔS to a positive value following an initial fall to a negative value under 5–15% PEG6000 stresses. Furthermore, under 5–15% PEG6000 stresses, the compressive stress that the cells exerted increased with the PEG6000 concentration, as did the time it took for ΔS to rebound to a positive value. When the cells were subjected to 25% PEG6000 stress, ΔS dropped to a negative value, reached its maximum absolute value, and finally did not return to a positive value. And the cells exerted greater immediate compressive stress.

**Figure 4:**
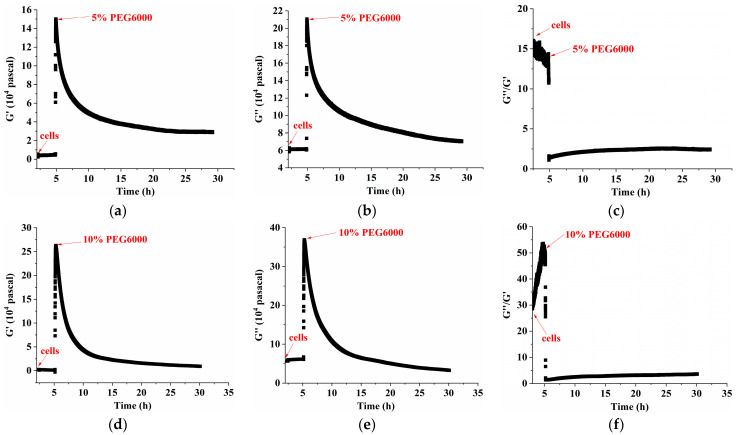

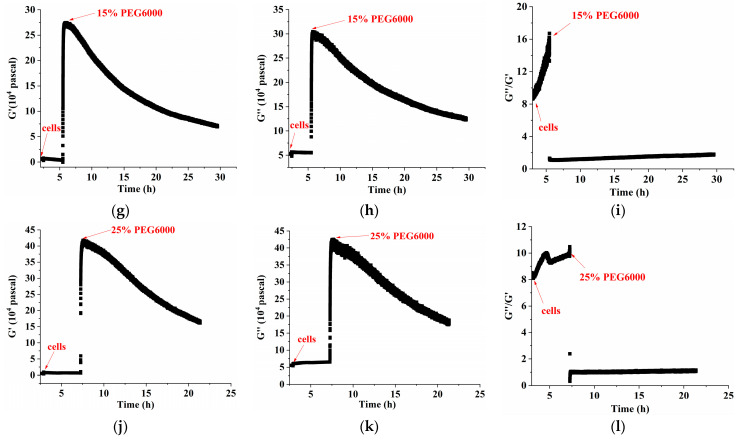
Changes in storage modulus (G′), loss modulus (G″), and loss tangent (G″/G′) of rice cells under different concentrations of PEG6000 stress monitored by 5 MHz DRPC technique. (**a**,**d**,**g**,**j**): G′, (**b**,**e**,**h**,**k**): G”, (**c**,**f**,**i**,**l**): G”/G′. (**a**–**c**): 5% PEG6000, (**d**–**f**): 10% PEG6000, (**g**–**i**): 15% PEG6000, (**j**–**l**): 25% PEG6000.

**Figure 5:**
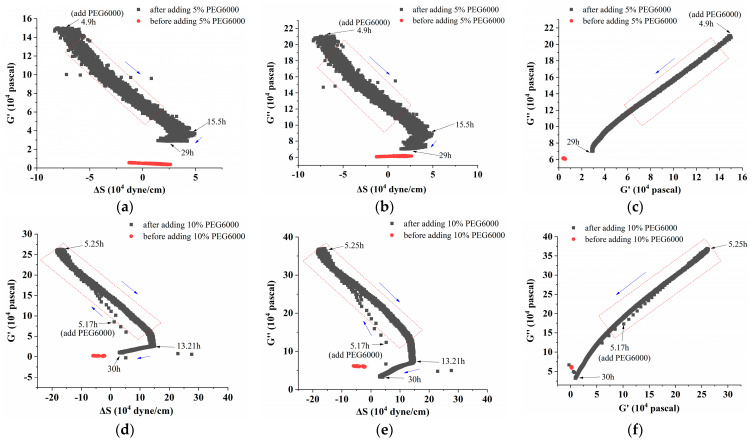

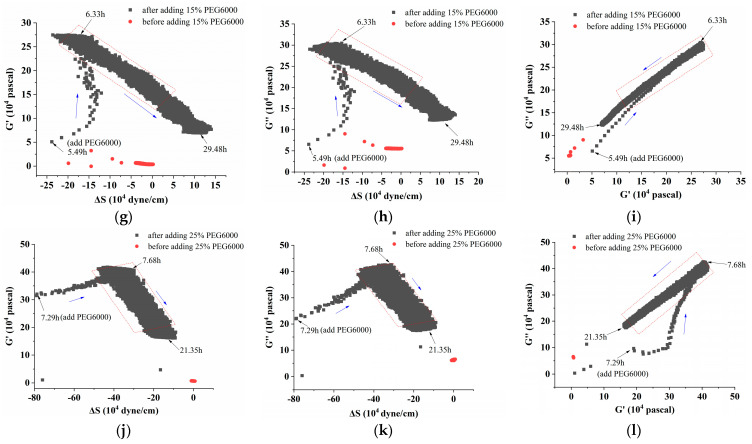
Correlations among the mechanical parameters of rice cells under the stress of different concentrations of PEG6000. Relationships between G′ and ΔS are shown in (**a**,**d**,**g**,**j**), G″ and ΔS are shown in (**b**,**e**,**h**,**k**), and G″ and G′ are shown in (**c**,**f**,**i**,**l**). PEG6000 concentrations: (**a**–**c**): 5%; (**d**–**f**): 10%; (**g**–**i**): 15%; (**j**–**l**): 25%. Black arrows showed the time when PEG6000 solutions were added and the following time points; while blue arrows showed the change trends of the curves.

Furthermore, curves showing the changes over time of storage modulus (G′), loss modulus (G″), and loss tangent (G″/G′) under 5–25% PEG6000 stresses were generated using equations (10) and (11), as shown in Figure 4. The DRPC technique also allows for the real-time monitoring of cell viscoelasticity. It is clear that the rice cells’ storage modulus (G′) and loss modulus (G″) showed a tendency of first increasing and then decreasing under 5–25% PEG6000 stresses. Moreover, under all the PEG6000 stresses, a shift in the cells’ state from a viscoelastic gel state to a more solid state was indicated by the general decrease in the loss tangent. The findings of the variations in the association among the mechanical parameters of rice cells at varying PEG6000 stress concentrations are shown in Figure 5. Generally speaking, the higher the contractile stress, the higher the viscoelastic moduli (G′, G″). There are linear regions for both ΔS~G′, ΔS~G″ for all PEG 6000 concentrations. However, we found that at 10% PEG6000, there was a brief and initial period during which both ΔS~G′ and ΔS~G″ deviated their linearities slightly; whereas at 15% PEG6000, initially, ΔS~G′, ΔS~G″ deviated their linearities more compared to 5% PEG6000 with even a brief period of positive ΔS~G′, ΔS~G″ relations implying that tensile stress was generated. At 25% PEG6000, pure positive ΔS~G′, ΔS~G″ relations occurred before they changed to negative relations, indicating that under strong drought stress, the protoplasts retracted from the cell wall resulting in dominated tensile stress by the stretching of the Hechtian strands. The total transfer of the positive ΔS~G′, ΔS~G″ to negative ΔS~G′, ΔS~G″ relations may imply the irreversible damage of the Hechtian strands. Therefore, the ΔS~G′, ΔS~G″ relations can indicate the sequences and types of forces generated and dictate the integrity of the plant cell wall–membrane–cytoskeleton continuum. The broken or damaged integrity can also be revealed by checking the G″~G′ relations. For 5% PEG6000, the G″~G′ curve obeyed linearity during the entire process of PEG6000 treatment. For 10–15% PEG6000, G″~G′ curves deviated linearity slightly in some regions. For 25%, the G″~G′ curve deviated linearity seriously during the initial time of the treatment, indicating damage to the wall–membrane–cytoskeleton continuum and its structures. The broken or damaged integrity can also be revealed by checking the G″~G′ relations. For 5% PEG6000, the G″~G′ curve obeyed linearity during the whole process of PEG6000 treatment. For 10–15% PEG6000, the G″~G′ curves deviated linearity slightly in some regions. For 25% PEG 6000, the G″~G′ curve deviated linearity seriously during the initial time of the treatment, indicating damage to the wall–membrane–cytoskeleton continuum and its structures.

### 3.2. Mechanical Changes of Rice Protoplasts under Different Concentrations of PEG6000 Stress

**Figure 6:**
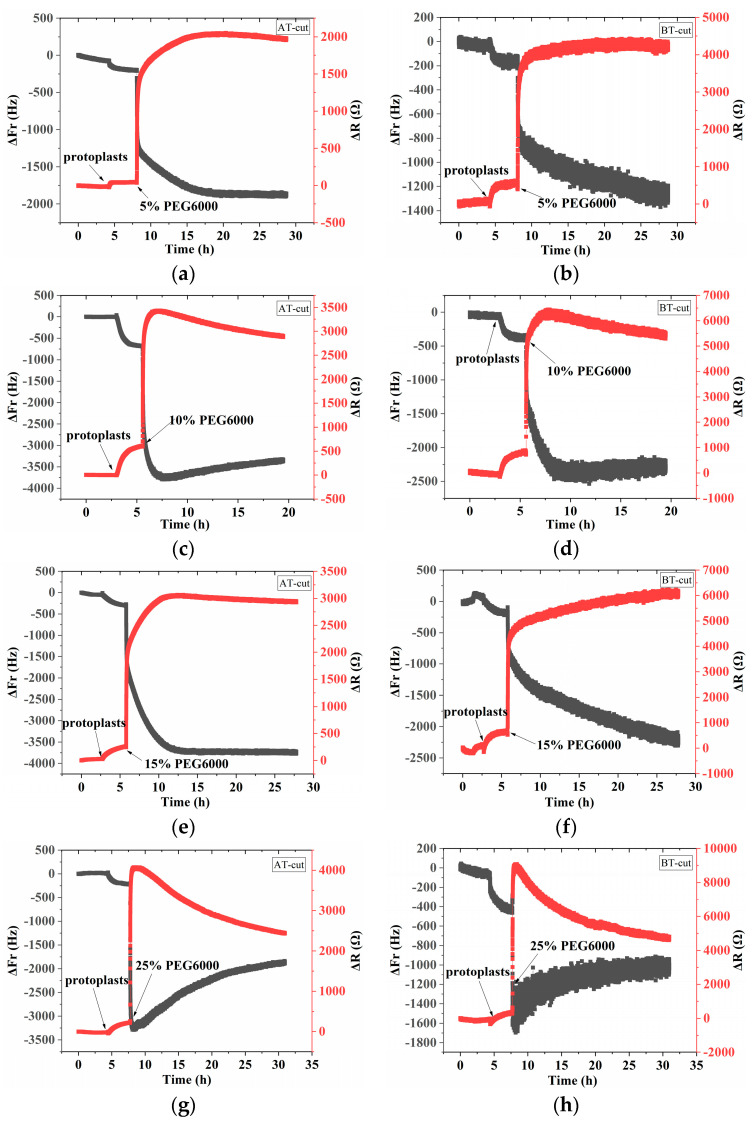
5 MHz DRPC tracking of frequency-resistance shifts brought about by rice protoplasts under varying PEG6000 stress concentrations: 5% PEG6000 in (**a**,**b**), 10% PEG6000 in (**c**,**d**), 15% PEG6000 in (**e**,**f**), and 25% PEG6000 in (**g**,**h**).

**Figure 7:**
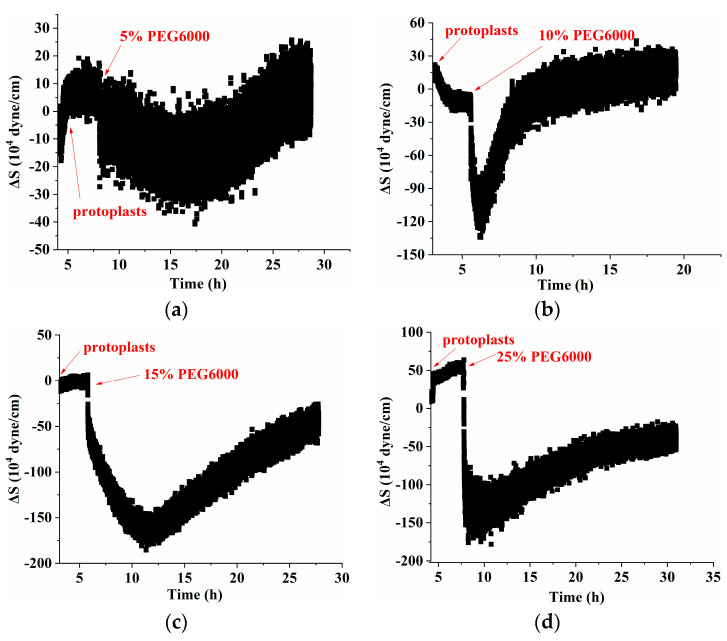
The 5 MHz DRPC tracking of stress (ΔS) variations produced by rice protoplasts at various PEG6000 stress concentrations. PEG 6000 concentrations: (**a**) 5%, (**b**) 10%, (**c**) 15%, and (**d**) 25%.

Figure 6 shows the frequency and resistance changes (ΔF and ΔR) of AT- and BT-cut quartz crystal chips during the adhesions of rice protoplasts followed by the treatments of different PEG6000 concentrations. Upon the addition of roughly 50,000 protoplasts, ΔF dropped and ΔR rose during the adhesions of the protoplasts. The protoplasts induced DRPC responses under 5–15% PEG6000 stresses, demonstrating an initial significant decrease in ΔF and increase in ΔR. Then, they became relatively stable. Compared to walled cells which showed dramatic rebounds of both ΔF and ΔR after their initial drop and increase (cf. Figure 2), wall-free protoplasts lacked similar rebounds. Under the treatment of 25% PEG6000, the protoplasts induced DRPC responses, showing significant recoveries after their dramatic initial drop and increase of ΔF and ΔR. Based on the DRPC concept, the stress variations (ΔS) produced by protoplasts under 5–25% PEG6000 stresses were calculated using Equation (9), which are presented in Figure 7. The stress ΔS produced by protoplasts under 5–15% PEG6000 stresses first dropped to negative values and then progressively recovered, suggesting that protoplasts first produced gradually increased compressive stress that gradually reduced over time. The stress ΔS generated by protoplasts declined to negative levels and did not fully recover under 25% PEG6000 stress. This suggests that under 25% PEG6000 stress, protoplasts maintain their contractile state while continually producing compressive stress.

**Figure 8:**
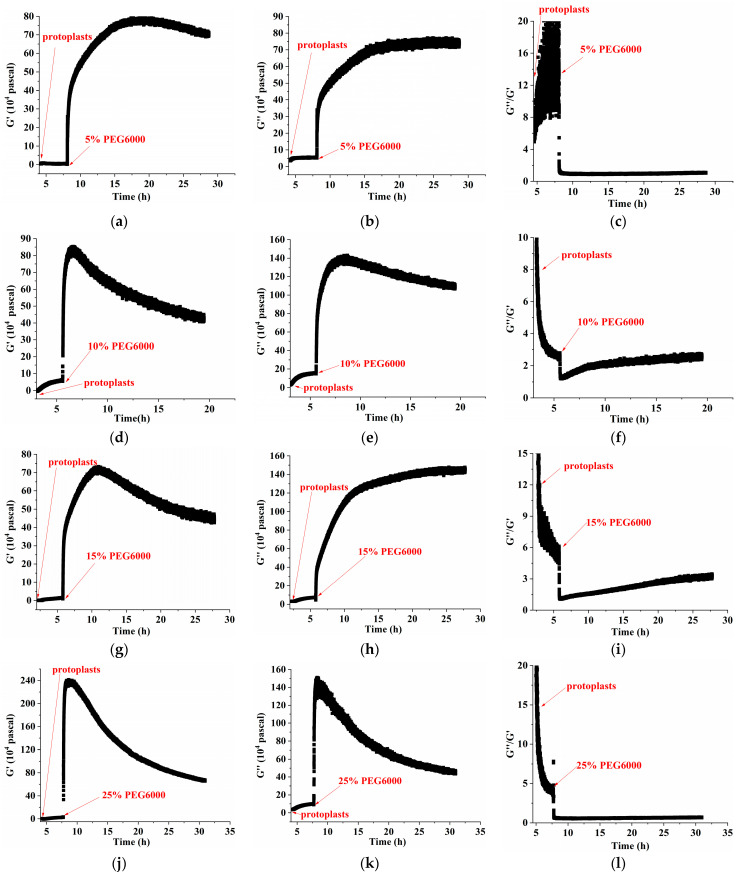
Variations in rice protoplasts’ storage modulus (G′), loss modulus (G″), and loss tangent (G″/G′) under stresses of different PEG6000 concentrations tracked using 5 MHz DRPC technique. G′: (**a**,**d**,**g**,**j**), G”: (**b**,**e**,**h**,**k**), G″/G′: (**c**,**f**,**i**,**l**). PEG6000 concentrations: (**a**–**c**): 5%; (**d**–**f**): 10%; (**g**–**i**): 15%; (**j**–**l**): 25%.

The protoplasts’ viscoelastic parameters were also calculated using Equations (10) and (11). We present the time-dependent curves of the protoplasts’ storage modulus (G′), loss modulus (G″), and loss tangent (G″/G′) under 5–25% PEG6000 stresses in Figure 8. The results show that, under 5% PEG6000 stress, protoplasts first became stiffer and then softened, changing from a viscoelastic gel-like state to a more solid state. The storage modulus (G′) of the protoplasts increased and then slightly decreased, the loss modulus (G″) increased and then remained almost constant, and the loss tangent (G″/G′) decreased and then remained essentially unchanged. The results show that under 10–15% PEG6000 stresses, protoplasts’ storage modulus (G′) and loss modulus (G″) increased and then decreased, while the loss tangent (G″/G′) decreased at first and then significantly increased. This suggests that protoplasts first stiffen and then significantly soften, moving from a viscoelastic gel-like state to a more solid state and finally reversing to a more fluid-like state.

**Figure 9:**
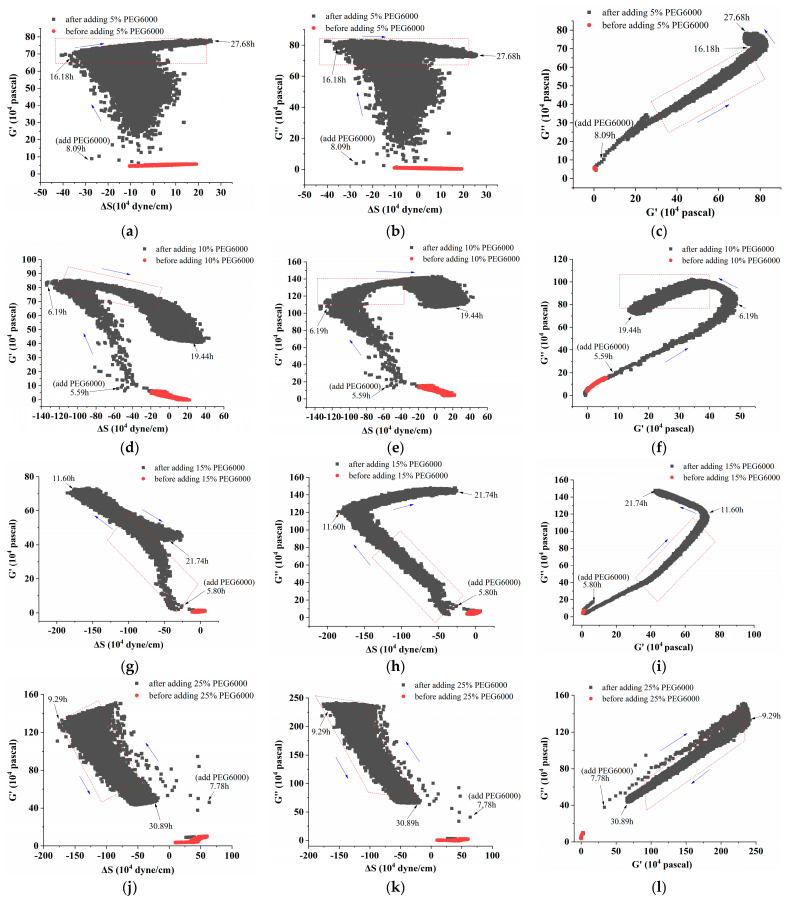
Correlations among the mechanical parameters of rice protoplasts under stresses of different concentrations of PEG6000. Relationships between G′ and ΔS are shown in (**a**,**d**,**g**,**j**), G″ and ΔS are shown in (**b**,**e**,**h**,**k**), and G″ and G′ are shown in (**c**,**f**,**i**,**l**). PEG6000 concentrations: (**a**–**c**): 5%; (**d**–**f**): 10%; (**g**–**i**): 15%; (**j**–**l**): 25%. Black arrows showed the time when PEG6000 solutions were added and the following time points; while blue arrows showed the change trends of the curves.

Under the stress of 25% PEG6000, the storage modulus (G′) and loss modulus (G″) of the protoplasts decreased more obviously after their initial increasing; and the loss tangent (G″/G′) decreased continually toward a value close to 0 and then stayed almost unchanged, indicating that the protoplasts transformed to a nearly solid-like state and remained unchanged. The correlated changes in the mechanical parameters of rice protoplasts under the stress of different PEG6000 concentrations are displayed in Figure 9. Here, for rice protoplasts, the ΔS~G′, ΔS~G″ relations are more complicated compared to rice cells (Figure 5). There are only limited regions where ΔS~G′, ΔS~G″ are linear. These curves are more like the ΔS~G′, ΔS~G″ relations obtained with human umbilical vein endothelial cells (HUVECs) during their adhesions under different ligand densities of adhesion molecules in that both tensile and contractile stresses were observed [31]. Under the action of different concentrations of PEG6000, in most situations, both contractile and tensile stresses also coexist in rice protoplasts, and the dynamic disassembly and assembly of cytoskeletons change the viscoelastic moduli of the protoplasts. Overall, the linearities of the G″~G′ relationships are much worse than those of the rice cells. These complex relationships may also suggest that the structural integrity of protoplasts is not as good as that of walled cells.

### 3.3. Comparison of Mechanical Properties of Rice Cells and Protoplasts under Different Concentrations of PEG6000 Stress

**Figure 10:**
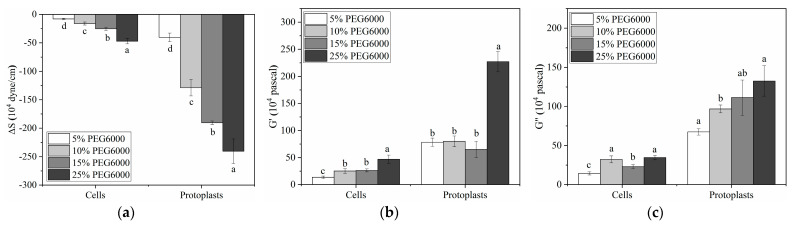
Comparisons of the mechanical properties of protoplasts and rice cells at varying PEG6000 stress concentrations. (**a**): ΔS, (**b**): G′, (**c**): G″. All tests were performed with three technical replicates. The significance is marked with letters. For variables with the same letters, the difference between the means is not statistically significant.

As illustrated in Figure 10, we contrasted the maximal force and moduli changes generated by cells and protoplasts prior to and following a 5–25% PEG6000 stress. Compared to cells, protoplasts show larger maximum changes in force and moduli before and after being subjected to the stress under the same PEG6000 concentrations. Furthermore, the stress produced by protoplasts and cells both increase with the increase in PEG6000 concentration (Figure 10a). For moduli G′ and G″, they do not appear to follow the predictable patterns, although there are overall increasing trends for both G′ and G″ with the increase in PEG6000 concentration (Figure 10b,c), suggesting a more complicated influence on moduli fluctuations. The mechanical characteristics of rice cells and protoplasts at various PEG6000 stress concentrations were then subjected to linear regression analysis, as shown in Table 2. Overall, there are stronger relationships among the mechanical properties of rice cells than protoplasts for limited time regions; this is another indication that the integrity of walled cells is much better than that of the well-free protoplasts.

### 3.4. Morphological Changes in Rice Cells and Protoplasts under Different Concentrations of PEG6000 Stress

**Figure 11:**
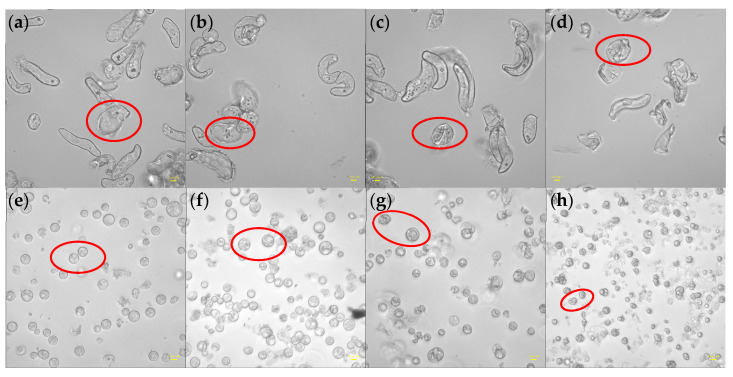
Morphologies of rice cells and protoplasts under the stress of different PEG6000 concentrations (scale bar: 10 μm). (**a**–**d**): Cells; (**e**–**h**): Protoplasts. (**b**,**f**): 5% PEG6000, (**c**,**g**): 15% PEG6000, (**d**,**h**): 25% PEG6000, and (**a**,**e**): blank control. Red circles showed representative cells under differnt statuses.

Using an Olympus inverted fluorescent microscope, the morphological changes of rice cells and protoplasts adhering on laser confocal plates before and one hour after the additions of different concentrations of PEG6000 were detected, as shown in Figure 11. Both cells and protoplasts in their first states (Figure 11a,e) showed strong, well-formed structures. The morphologies of both cells and protoplasts appeared to be largely intact under 5% PEG6000 stress (Figure 11b,f), with many tiny particles developing within the cells and abnormalities along the protoplast membranes with disordered cytoplasmic structures. Under the stress of 15% PEG6000, both cells and protoplasts collapsed (Figure 11c,g). Ultimately, both cells and protoplasts experienced significant dehydration and distortion under 25% PEG6000 stress (Figure 11d,h). During our microscopic observations of cells’ morphologies under different concentrations of PEG6000 stress, we observed cytoplasmic streaming in the cells under 5–15% PEG6000 stress, but not under 25% PEG6000 stress, indicating that the cells maintained good viability under 5–15% PEG6000 stress but lost their viability under 25% PEG6000 stress.

## 4. Discussion

In this study, we investigated the mechanical characteristics of rice cells and protoplasts under PEG6000-simulated drought stress by our newly developed DRPC technique, and the results showed that the mechanical responses of the cells and protoplasts during the drought stress adaptation varied. The cells displayed a brief compressive stress that was succeeded by a slow onset of tensile stress (cf. Figure 3). Cell dehydration was ascribed for this, as it caused a sharp drop in turgor pressure and momentary compressive stress. Tensile stress was produced by the stretching of Hechtian strands between the cell wall and plasma membrane in protoplasts, which had shrunk as a result of water loss. According to Elizabeth S. Haswell et al.‘s description of the molecular pathways by which plants perceive and react to osmotic challenges, most protoplasts separate from the cell wall during the process of plasmolysis, but the Hechtian strands still remain to connect the plasma membrane and the cell wall [37]. Thus, membrane tension can be increased by the protoplast’s expansion as well as its shrinking. On the other hand, protoplasts lacking a cell wall do not produce significant tensile stress under the same stress circumstances because of the absence of the Hechtian threads. The volume impact caused by drought stress may be the reason for the initial increase and subsequent decrease in compressive stress produced by protoplasts (cf. Figure 7). Traction force microscopy was used by Liu Y et al. [38] to quantify the traction force that animal cells exert on a substrate. They discovered that, in the presence of excessive osmolarity, traction force first increased and subsequently declined, with cell traction force fluctuating in response to volume variations. Michael P. Murrell et al.’s research [39] revealed that lipid droplets scattered over pliable substrates generate strong traction forces. Using a micro-rheometer, Durand-Smet et al. [40] examined the rheology of individual protoplasts and animal cells and discovered that both showed mild power-law rheology. Therefore, these research findings provide scientific justification for our parallel comparisons between protoplasts and animal cells. During the late stages of stress, we observed that protoplasts showed a progressive decrease in compressive stress (cf. Figure 7), indicating a sluggish recovery of their volume. This conclusion is in line with the findings of Chloé Roffay et al. [41], who observed incomplete restoration even after several hours and sluggish volume recovery in cells under acute hypertonic stress. The differences in stress resistance between walled cells and protoplasts lacking the cell wall may also be reflected in the varying response rates of cells and protoplasts to the same stress. Under unfavorable stress conditions, cells react quickly, but protoplasts react more slowly (cf. Figure 3 and Figure 9).

Moreover, a substantial amount of evidence suggests that abiotic stresses cause the cytoskeletal network to reorganize [42,43,44,45]. The tensegrity model [46] states that actin filaments dominate tensile stress and microtubules dominate compressive stress. According to An-Shan Hsiao et al. [47], rice cells that express intrinsic disordered proteins (RePRPs) attach to actin filaments in water-deficient circumstances, thereby decreasing the amount of actin filaments and reorienting the microtubule network through the binding of microtubule proteins. This implies a reduction in the tensile stress dominated by actin filaments, and ΔS is mainly dominated by compressive stress produced by microtubules. Actin filaments in plant cells have been seen to depolymerize and subsequently reassemble under high osmotic stress [48,49], suggesting a progressive restoration of actin filament-dominated tensile stress and a gradual reduction in compressive stress. Consistent with our observations under the microscope, we observed that the changes in the mechanical properties of cells and protoplasts under 25% PEG6000 stress were similar (cf. Figure 3d and Figure 9d). This suggests that at this concentration, both cells and protoplasts may experience strong contractions, which could lead to possible breaking of Hechtian strands and/or the disruption of the cytoskeletal network, resulting in irreversible damages to these cellular structures and neither the cells nor the protoplasts could withstand the 25% PEG6000 stress. We observed by an optical microscope that the cells and protoplasts underwent severe deformations at a 25% PEG6000 concentration (cf. Figure 11d,h), which supports our cytomechanical test results. It has been reported that the stress generated by cells is positively correlated with turgor pressure and cell radius, and negatively correlated with cell wall thickness [21]. Assuming that the turgor pressures generated by rice cells and wall-removed protoplasts are the same, as the cell wall thickness is much larger than that of the plasma membrane cortex, the measured stress generated by protoplasts should be much greater than that of the walled cells. This was exactly observed as shown in Figure 10. In addition, under the same stress conditions, the maximum stress generated by cells is greater than that of protoplasts, and both increase with the increase in stress concentration (cf. Figure 10a). These are in line with the fact that the observed changes in cell morphologies also increased with the increase in stress concentration, further confirming the reliability of DRPC technology.

Under the stress of 5–25% PEG6000 concentrations, we investigated and compared the changes in cellular viscoelasticity of protoplasts and rice cells. The findings demonstrated a quick increase and subsequent decrease in the cell storage modulus (cf. Figure 4). This could be connected to how drought stress causes plant cells to produce reactive oxygen species (ROS) and Ca^2+^. According to related research, ROS and peroxidases can form crosslinks with glycoproteins in the cell wall and phenolic chemicals, which can cause the cell wall to harden. When ROS levels stay high, OH^−^ radicals are formed, which leads to polymer disintegration and the loosening of the cell wall due to swelling proteins [3,50], just as we observed that many granular substances were produced inside the cells under PEG6000 stress, while they were absent in the protoplasts (cf. Figure 11). This indicates the regulatory role of substances such as ROS and peroxidase in the cell wall’s viscoelasticity. Furthermore, plant cells have the ability to create acidic microdomains, which induce cell wall loosening, or Ca^2+^-pectic acid crosslinking, which promotes cell wall hardness [51]. Because of the quick production of ROS and Ca^2+^, rice cells under drought stress quickly stiffen and then soften, illustrating the cell wall’s malleability in the process of plant cell resistance [2]. Moreover, it has been reported that bacterial cells treated with strong osmotic pressure for 30 s thicken their cell wall, which thins down an hour later [52]. This process can also occur in plant cells, causing a brief stiffening and then relaxing accompanied with a drop in ΔS and then an increase in ΔS. According to our findings, protoplasts’ storage modulus progressively rises before falling (cf. Figure 8), which may be as a result of cytoskeletal and volume modifications brought about by drought stress. After applying osmotic stress to stem cells, Ming Guo et al. [53] found a strong and reliable correlation between stem cell stiffness and cell volume, that is, as cell volume falls, cell stiffness rises. The study by Leah Ginsberg et al. [17] demonstrates that the cytoskeleton plays a major role in determining the stiffness and dissipation energy of tobacco cells during indentation. They discovered that the MT network contributes nearly twice as much stiffness as the AF network. According to the shift in the loss tangent, cells experience a tendency to transition to a more solid state when under drought stress, whilst protoplasts show a tendency to move to a more fluid-like state. This could imply that plant cells’ inflexible cell wall construction is essential for their ability to withstand environmental stress. Furthermore, compared to protoplasts, our findings show much stronger links among the mechanical parameters of rice cells (cf. Figure 5 and Figure 9 and Table 2). The existence of a cell wall would provide plant cells with a more integrative structure to resist and adapt to various abiotic stresses. This emphasizes the significance of the intact cell wall–plasma membrane perception of physical stresses to some extent [54].

It is well known that living cells are not homogeneous, i.e., different cellular structures have different viscoelastic moduli, as reported in [53] that the bulk, cortical, and cytoplasmic moduli of A7 cells all increased as cell volume decreased under external osmotic pressures; however, their moduli are in the following order: bulk > cortical > cytoplasmic. For plant cells with a cell wall, it is even more non-homogeneous. Moreover, the viscoelastic moduli are dynamic, i.e., a spatiotemporal dynamic monitoring technique is required to capture the whole viscoelastic picture; however, right now, no single rheological technique offers the capability. One strategy is to combine several techniques targeting different cellular structures as performed in [53], but only end-points results were reported and dynamic information was still missing. The DRPC technique allows for measuring the dynamic viscoelastic moduli of living cells in compact contact with the DRPC chips based on the assumption that the cells are homogeneous and the thickness of the cells is much larger than that of the penetration depth of the acoustic wave to the cells. Under the stress of PEG6000, the organelles and cellular structure would undergo dynamic remodeling resulting in dynamic changes in their distances to the chips; and the magnitudes of these changes depend on the concentrations of PEG6000. Therefore, the DRPC technique may not detect the same cellular structures and/or the detection sensitivity would change with a change in the PEG6000 concentration. This gives an explanation for why G′ and G″ do not vary significantly as the percentages of PEG6000 increase (cf. Figure 10b,c). Moreover, unlike animal cells which are adherent with known mechanosensing molecule integrin, plant cells cannot adhere to the substrates by themselves, and no known mechanosening molecule like integrin has been found and confirmed so far. In this work, we only modified the DRPC substrates with positively charged PDADMAC, promoting the adhesions of both rice cells and protoplasts, which may limit the detection sensitivity and linear range to cytoskeleton and other cellular structures governing cell viscoelasticity; this can be improved in a future study by exploring and modifying molecules or materials on the DRPC chips, which can interact specifically with potential mechanosensng molecules or receptors in living plant cells.

**Figure 12:**
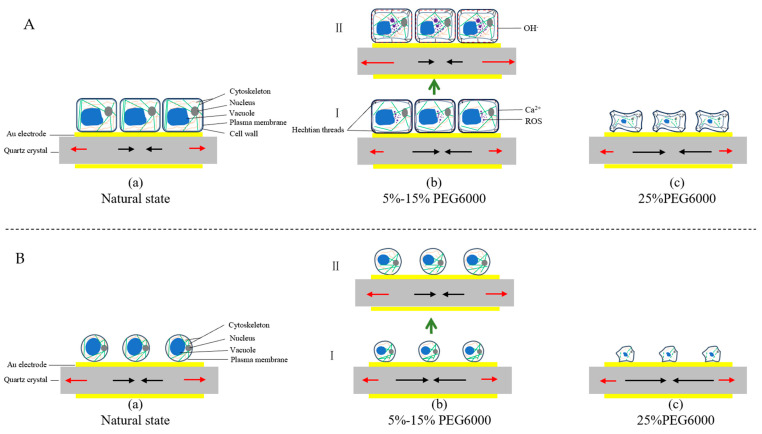
Schematic illustrations of the structural, morphological, and metabolic changes in rice cells and protoplasts under drought stress of PEG6000 and the resulting cytomechanical responses. Scheme (**A**): rice cells, Scheme (**B**): rice protoplasts. (**a**): natural state, (**b**): 5–15% PEG6000, (**c**): 25% PEG6000. (**I**) Initial status after being subjected to the treatments of 5%-15% PEG6000. (**II**) Subsequent status following status I. Red and black arrows represent tensile and compressive stresses respectively.

To summarize our work, we present Figure 12 to show the dynamic changes in both the cytomechanical parameters (cells’ generated forces and viscoelastic moduli) and the cellular structures, morphologies and metabolites of rice cells (Figure 12A) and protoplasts (Figure 12B) under different degrees of drought stress to illustrate the biosensing principles of DRPPC to drought stress. Through DRPC technology, we successfully monitored the dynamic changes in the cells’ generated forces and viscoelastic moduli of rice cells and protoplasts under drought stress reflecting the dynamic changes of plant cell structure, morphology, and metabolism. As shown in Figure 12(Aa,Ba), under normal culture conditions without environmental stresses, both rice cells and protoplasts are in their natural status (forces are in balance) maintaining their intact internal structures in good order. When the cells were subjected to drought stress, the cells showed drought resistance and adaptive responses by changing their mechanical properties of cellular forces and viscoelasticity. Under the tolerable stress of 5–15% PEG6000 (Figure 12(Ab)), the cells initially generated compressive stress due to protoplast contraction, and then tensile stress gradually became dominant, possibly due to the stretching of the Hechtian threads. In addition, the disassembly and reassembly of actin microfilaments may also account for the initial compressive stress followed by the reversal of tensile stress. The cell wall hardened instantly due to the rapid production of ROS and Ca^2+^ under drought stress (Figure 12(AbI)); as the duration of PEG6000 treatments increased, ROS continued to increase, and OH^−^ was produced, leading to the cleavage of the cell wall and resulting in wall softening (Figure 12(AbII)). Under intolerable drought stress of 25% PEG6000, the cells crumpled to produce compressive stress only; cellular structures including Hechtian threads and cytoskeletons were probably irreversibly damaged (Figure 12(Ac)).

Under drought stress of 5–15% PEG6000, rice protoplasts exhibited dynamic mechanical responses in force and viscoelasticity mainly due to the changes in the cellular structure of cytoskeletons and morphologies (Figure 12(Bb)). The disassembly and reassembly of actin microfilaments is the main factor leading to the initial increase and then decrease in the protoplasts’ generated compressive stress. Moreover, the reduced size under drought stress and its gradual recovery of protoplasts is another reason for the dynamic changes in the compressive stress observed. Since protoplasts lack cell wall and Hechtian threads, no obvious tensile stress was observed. The reduction in protoplast volume due to water loss leads to protoplast hardening, and the subsequent volume recovery leads to protoplast softening. Under intolerable drought stress of 25% PEG6000, the protoplasts instantly generated compressive stress accompanied with rapid increases in cells’ viscoelastic moduli; dramatic change in the cell’s morphology was observed, indicating irreversible damages of cellular structures including plasma membrane and cytoskeleton (Figure 12(Bc)).

## 5. Conclusions

In order to dynamically and non-destructively determine the mechanical characteristics of rice cells and protoplasts under 5–25% PEG6000 stress, this study used our newly developed Double Resonator Piezoelectric Cytometry (DRPC) approach. The outcomes demonstrated that under 5–15% PEG6000 stress, the mechanical performance of cells and protoplasts varied considerably. At first, compressive stress was seen in both cells and protoplasts; however, cells generated compressive stress at a greater rate than protoplasts did. Tensile stress was then slowly observed in the cells; however, it was almost absent from the protoplasts. Furthermore, there was an early rise and then a decline in the elastic modulus and loss modulus of cells and protoplasts. While the loss tangent of protoplasts showed an initial decrease followed by an increase, the loss tangent of cells generally declined. Similar alterations were seen in the mechanical characteristics of both the cells and protoplasts under the stress of 25% PEG6000. It is worth noting that the maximum stresses produced by cells under the stress of 5–25% PEG6000 were smaller than that of the protoplasts, and the maximum stresses produced by both of them increased with the increase in PEG6000 concentration. Moreover, the morphological changes of rice cells and protoplasts observed by optical microscopy further confirmed the results of DRPC. In summary, the changes in the cells generated stress and viscoelastic moduli of rice cells and protoplasts under the stress of different concentrations of PEG6000 reflect the differences in mechanical properties of rice cells before and after wall removal. The results show that DRPC technology is expected to provide biomechanical information for studying the changes in plant cells under various unfavorable stress conditions and provides a novel tool for the study of plant stress physiology at the cellular level.

## Figures and Tables

**Table 1:** A comparison of the main functions of existing plant cell mechanics methods.

Method/Instrument	Cell Viscoelasticity	Cellular Force	Simultaneous Measurement of Cellular Force and Viscoelasticity	Nondestructive Long-Term	Single Cell	Cell Cluster
Pico gauges	−	+	−	−	+	−
Indentation techniques	+	−	−	−	+	−
Atomic force microscopy, AFM	+	+	−	−	+	−
Brillouin scattering microscopy, BSM	+	−	−	+	+	−
Quartz Crystal Microbalance, QCM	+	−	−	+	+	+
Parallel plate rheometer, PPR	+	+	−	−	+	−
Microfluidic—Cell mechanics techniques	+	+	−	+	+	+
Double resonator piezoelectric cytometry, DRPC	+	+	+	+	+	+

Note: (+) With the function. (−) Without the function.

**Table 2:** Relationship among mechanical parameters of rice cells and protoplasts at various PEG6000 stress concentrations as determined by linear regressions.

	Concentration of PEG6000	G′~ΔS			G″~ΔS			G″~G′		
		Slope	R2	Time Region (h)	Slope	R2	Time Region (h)	Slope	R2	Time Region (h)
cells	5%	−0.8577	0.9739	5.03–13.6	−0.8972	0.9688	5.03–13.6	1.0482	0.9981	5.03–13.6
	10%	−0.6484	0.9927	5.17–10.43	−0.7641	0.9784	5.17–10.43	1.1838	0.9947	5.17–10.43
	15%	−0.6054	0.9764	5.63–18.82	−0.4849	0.9601	5.63–18.82	0.8062	0.9961	5.63–18.82
	25%	−0.8934	0.9146	7.31–21.36	−0.8029	0.8758	7.31–21.36	0.9157	0.9939	7.31–21.36
protoplasts	5%	−0.1944	0.7186	16.02–28.7	0.1184	0.7316	16.02–28.7	0.7932	0.9839	8.19–16.02
	10%	−0.1340	0.8998	6.23–8.64	0.2411	0.8425	6.23–8.64	1.2517	0.9330	8.72–19.42
	15%	−0.4370	0.9468	5.52–9.72	−0.6866	0.9684	5.52–9.72	2.2203	0.9866	6.00–10.7
	25%	−1.5380	0.9128	9.15–29.64	−0.7824	0.8732	9.15–29.64	0.5317	0.9923	7.96–29.58

## Data Availability

The authors confirm that the data supporting the findings of this study are available within the article.
